# Multipoint Tissue Circulation Monitoring with a Flexible Optical Probe

**DOI:** 10.1038/s41598-017-10115-5

**Published:** 2017-08-29

**Authors:** Yoko Tomioka, Shintaro Enomoto, Jian Gu, Akiko Kaneko, Itsuro Saito, Yusuke Inoue, Taeseong Woo, Isao Koshima, Kotaro Yoshimura, Takao Someya, Masaki Sekino

**Affiliations:** 1 Department of Plastic Surgery, Graduate School of Medicine, University of Tokyo, Japan, Hongo 7-3-1, Bunkyo-ku, Tokyo 113-8655 Japan; 2 Department of Electrical Engineering and Information Systems, Graduate School of Engineering, University of Tokyo, Japan, Hongo 7-3-1, Bunkyo-ku, Tokyo 113-8655 Japan; 3 Department of Biomedical Engineering, Graduate School of Medicine, University of Tokyo, Japan, Hongo 7-3-1, Bunkyo-ku, Tokyo 113-8655 Japan; 4 Department of Plastic Surgery, Jichi Medical University, Japan, Yakushiji 3311, Shimotsuke-City, Tochigi 329-0498 Japan

## Abstract

Compromised circulation is a potential complication during the postoperative period following tissue transplantation. The use of a monitoring device allows physicians to detect compromised circulation immediately. Such monitoring devices need to be continuously usable, wearable, and area-detectable. However, existing devices fail to satisfy all of these requirements simultaneously. We developed a wearable, multipoint pulse wave-monitoring device. An array of reflective optical sensors implemented on a thin film substrate was used as a lightweight and flexible probe. As a model of tissue transplantation, an inguinal flap in a Wistar rat was dissected and freed from all subcutaneous tissue. By ligating the artery or vein, ischemia or congestion was induced in the tissue. In a human study, ischemia or congestion was induced in the palm by pressing the feeding artery or cutaneous vein, respectively. The amplitude of the pulse wave was evaluated using the power spectrum of Fourier transformed signals. Pulse wave amplitude significantly decreased under compromised circulation in both animal and human models. Moreover, we accomplished 1 week of continuous wireless monitoring in healthy subjects. These results demonstrated the potential utility of the developed device in postoperative blood-flow monitoring to improve the rescue rate of transplanted tissue.

## Introduction

Tissue transplantation is an effective technique in modern medicine for the cosmetic and functional reconstruction of tissue defects. It is now possible to reconstruct tissue after resection for cancer in the breast and neck, or to reconstruct limbs and fingers after trauma^1^ or due to congenital diseases. Because of the increasing expectancy that therapy should improve patient quality of life, plastic reconstruction using free flaps is rapidly developing^2, 3^. Although very high success rates of 90–99% have been reported for procedures performed by experienced teams^4–8^, vascular thrombosis remains a major complication of these transplants, mostly via venous thrombosis occurring within the first 48 hours after surgery^7, 9, 10^. Therefore, early detection and timely salvage surgery to resolve vascular occlusion in the postoperative period are required^9, 11, 12^ to prevent tissue necrosis^8, 13^.

Conventional observation after tissue transplantation involves the periodic manual checking of skin colour, temperature, capillary refilling, and bleeding. These approaches are time and energy consuming^14^ for both the patients and medical staff. Moreover, a high level of professional skill and experience are required to diagnose compromised circulation after transplantation based on the results of these assessments, and mistakes are possible. Lags between the onset of compromised circulation and its detection are common, and reduce the rescue rate of the transplanted tissue. Compromised circulation occurs over the entire flap if a thrombus appears in the pedicle of the nutrition vasculature, while partial flap failure occurs if a thrombus appears in branched vessels. Partial flap failure can progress to entire flap failure in certain conditions. Emergency salvage surgery is required in these cases.

With the above points in mind, a new method is required to deal with these issues. A device designed to solve these problems should have a number of functions, such as blood flow monitoring, area-detection, portability, and the capacity for continuous monitoring. Area-detection with multi-point measurement is required to detect partially compromised circulation. Portability is also required to allow patients to ambulate freely. Finally, continuous measurement is necessary for the timely detection of flap failure. Several conventional devices have been reported to monitor blood flow, such as laser Doppler^15, 16^, laser speckle contrast imaging (LSCI)^17, 18^, thermography^19^, blood glucose analysis^20^, and implantable probes^21–23^. In addition to these approaches, SpO_2_ monitoring^12, 24, 25^ and near infrared spectroscopy (NIRS) have been studied, but have limited functionality. Laser Doppler measures the direction and velocity of blood flow by capturing reflected light from red blood cells and assessing demodulated frequency shifts due to the Doppler effect. A handheld Doppler device has also been developed^13, 14, 26, 27^. Meanwhile, the LSCI captures superimposed Doppler shift from red blood cells, allowing blood-flow mapping over an extensive area^28^. However, the lasers used in such machines are not suitable for portable use due to their large size, and patients need to be undressed for the examination.

Flap failure can also be detected using thermography by measuring temperature decreases in the flap^19, 29^. Thermography is widely used in medical check-ups, and it is therefore feasible to use such devices to monitor circulation in the flap. However, only ischemia has been detected in previous studies, and the skin must be exposed for successful examination^30^. Blood glucose measurement devices are compact and easy to use in flap check-ups, but require time and effort due to the requirement for periodical assessment. Implantable probes enable blood-flow to be continuously monitored, but introduce the risk of hematoma or infection at the surgical site.

To achieve continuous monitoring, a portable device is essential. Furthermore, to allow area mapping, an array of sensors should ideally be distributed over the curved surface of the transplanted tissue. With the recent development of flexible electronic devices, it has become possible to implement multiple small sensors on a film substrate^31^. This technique is potentially useful in the multipoint measurement of blood-flow within the human body.

In this study, we evaluated a wearable pulse wave-monitoring device equipped with a flexible, multipoint sensor probe, and tested it to ensure that compromised circulation can be distinguished from normal blood-flow in both animal and human models. Throughout this article, pulse wave also refers to cardiac wave. Moreover, we tested the longevity of the device, and found that continuous monitoring was possible for 1 week.

## Results

### Pulse wave measurement in rats

**Figure 1:**
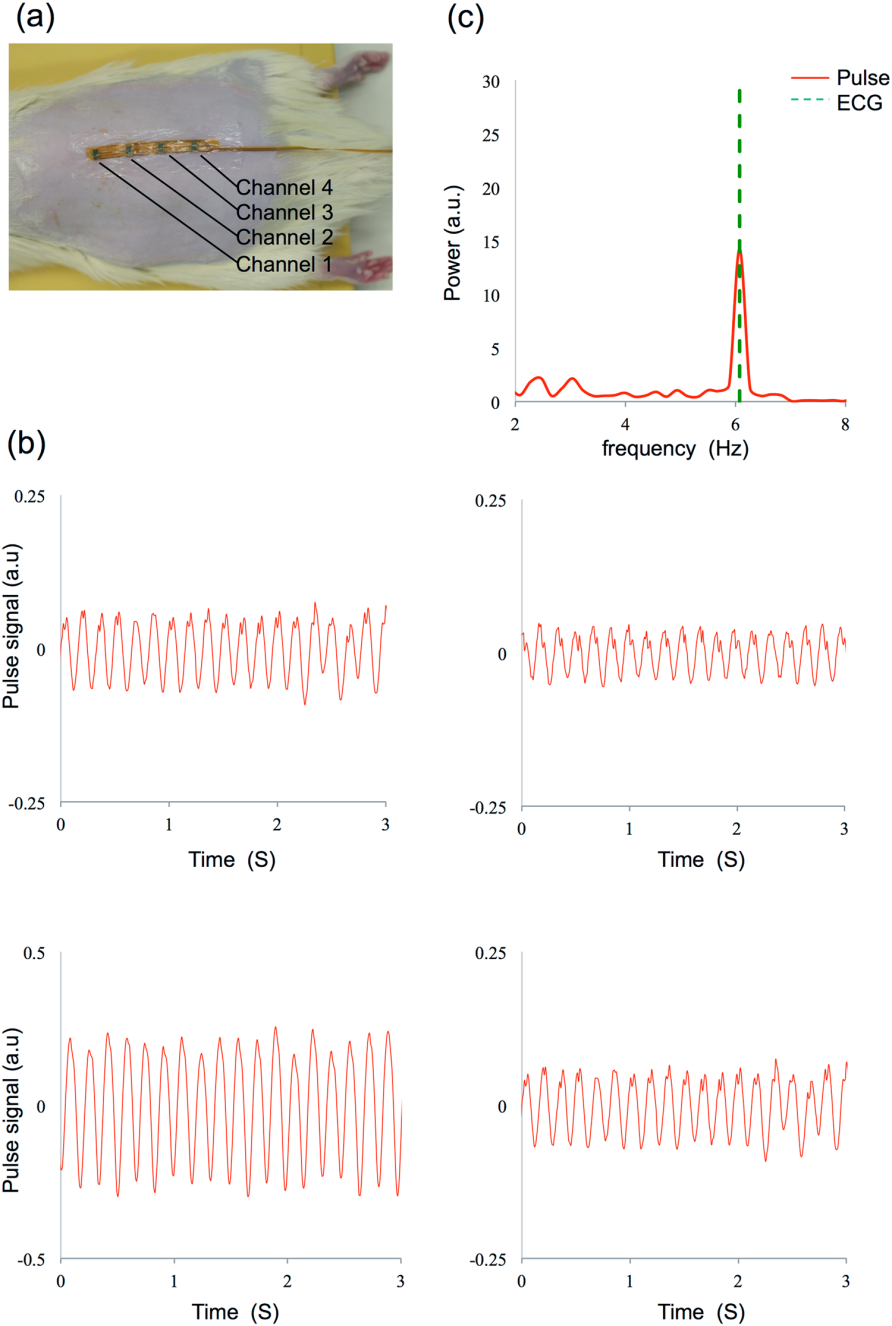
Measurement and analysis of multipoint microcirculation detection in rats. (**a**) Setup of the sensor probe. (**b**) Pulse wave signals obtained from all four channels (top left: channel 1, top right: channel 2, bottom left: channel 3, bottom right: channel 4) 5 s after activating the device. (**c**) Fast Fourier transform analysis of pulse wave signals and electrocardiogram (ECG) readings corresponding with one channel in (**b**). The peak frequency of the pulse wave and the ECG reading within the 2–8 Hz bandwidth are in synchrony.

Figure 1a shows the setup of the multipoint measurement device. Output signals of four channels are shown in Fig. 1b. We observed a clear pulse wave in all four channels. Results for one of the FFT analyses is given in Fig. 1c. The solid curve shows the spectrum of the signal measured by the sensor probe, and the dashed line shows the frequency of heart rate measured by ECG. The frequency of the maximum pulse power corresponded to the frequency of the heart rate measured by ECG, which indicated that we succeeded in measuring the pulse wave with the sensor probe.

**Figure 2:**
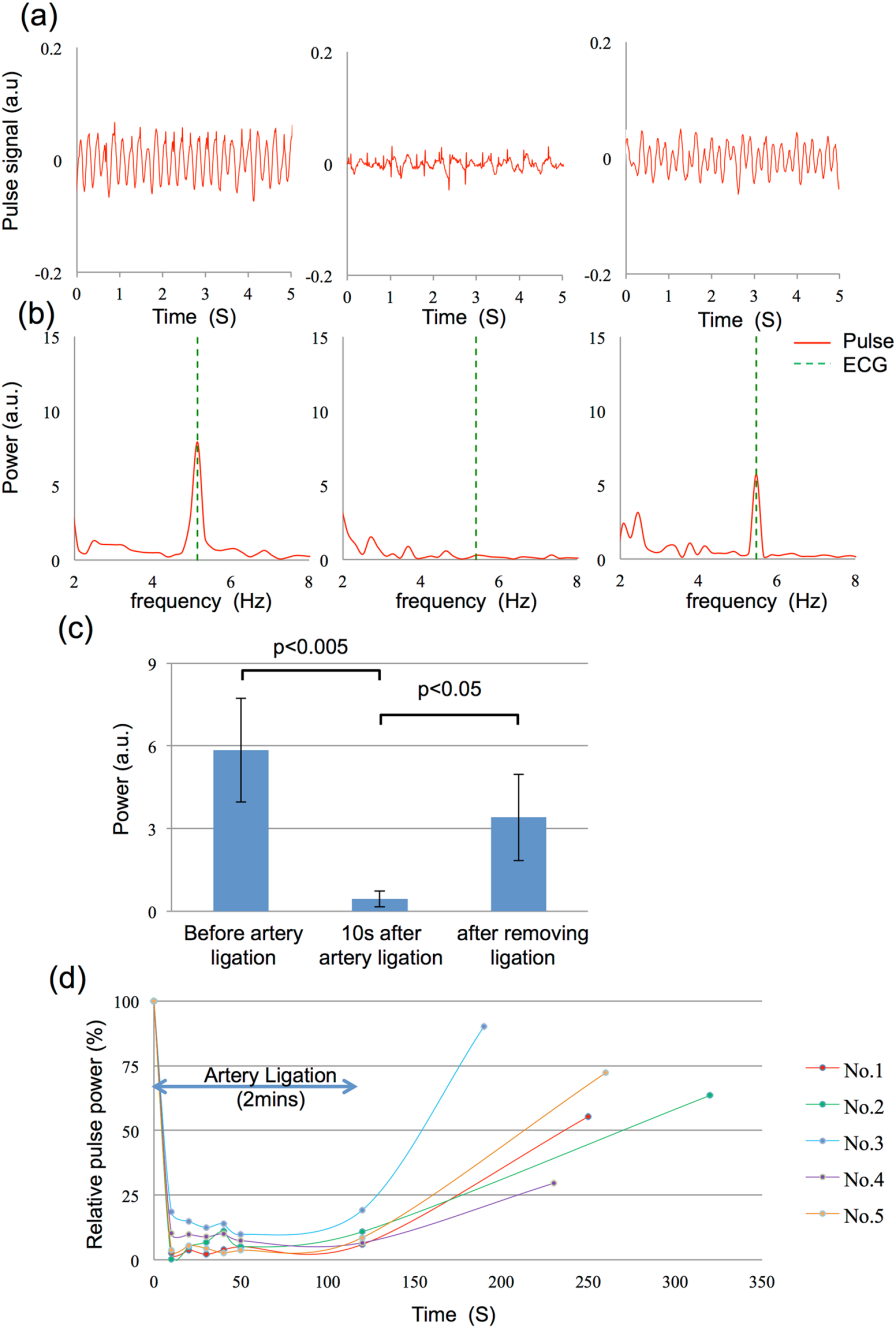
Measurement and analysis of microcirculation in the rat flap ischemia model. (**a**) Data measured before ligating the superior inferior epigastric artery (left), 5 s after ligation (center) and 3 min 20 s after releasing the ligation (right). (**b**) Spectrum of pulse wave signals and electrocardiography readings in (**a**). (**c**) *t*-test result of the data from five rats. Standard deviations in each group are $\pm$1.89 before ligation, $\pm$0.28 after ligation, and $\pm$1.57 after removing ligation. (**d**) Relative pulse power change with time. The initial value before ligation is set as a 100% reference.

Figure 2 shows the results of the ischemia test. The left, centre, and right panels in Fig. 2a show the signals obtained before artery ligation, 5 s after artery ligation, and 200 s after releasing the ligation, respectively. The pulse wave signal significantly decreased after ligation. FFT analysis results corresponding to each output signal in Fig. 2a are shown in Fig. 2b. The frequency of maximum pulse power corresponded to the heartbeat frequency measured by ECG. However, only low-amplitude pulse power was detected at the heartbeat frequency after ligation, as shown in Fig. 2b (centre). The *t*-test result of five rats is summarized in Fig. 2c. A significant difference (p < 0.05) was observed between ischemia and normal blood-flow conditions. The pulse power values of the five rats during the entire ischemia protocol are plotted in Fig. 2d. Pulse power in the normal state before artery ligation was treated as a reference. Pulse power steeply decreased for 10 s after ligation in all five rats. Relative pulse power did not exceed 20% during ligation, and recovered gradually to >30% after releasing the ligation.

**Figure 3:**
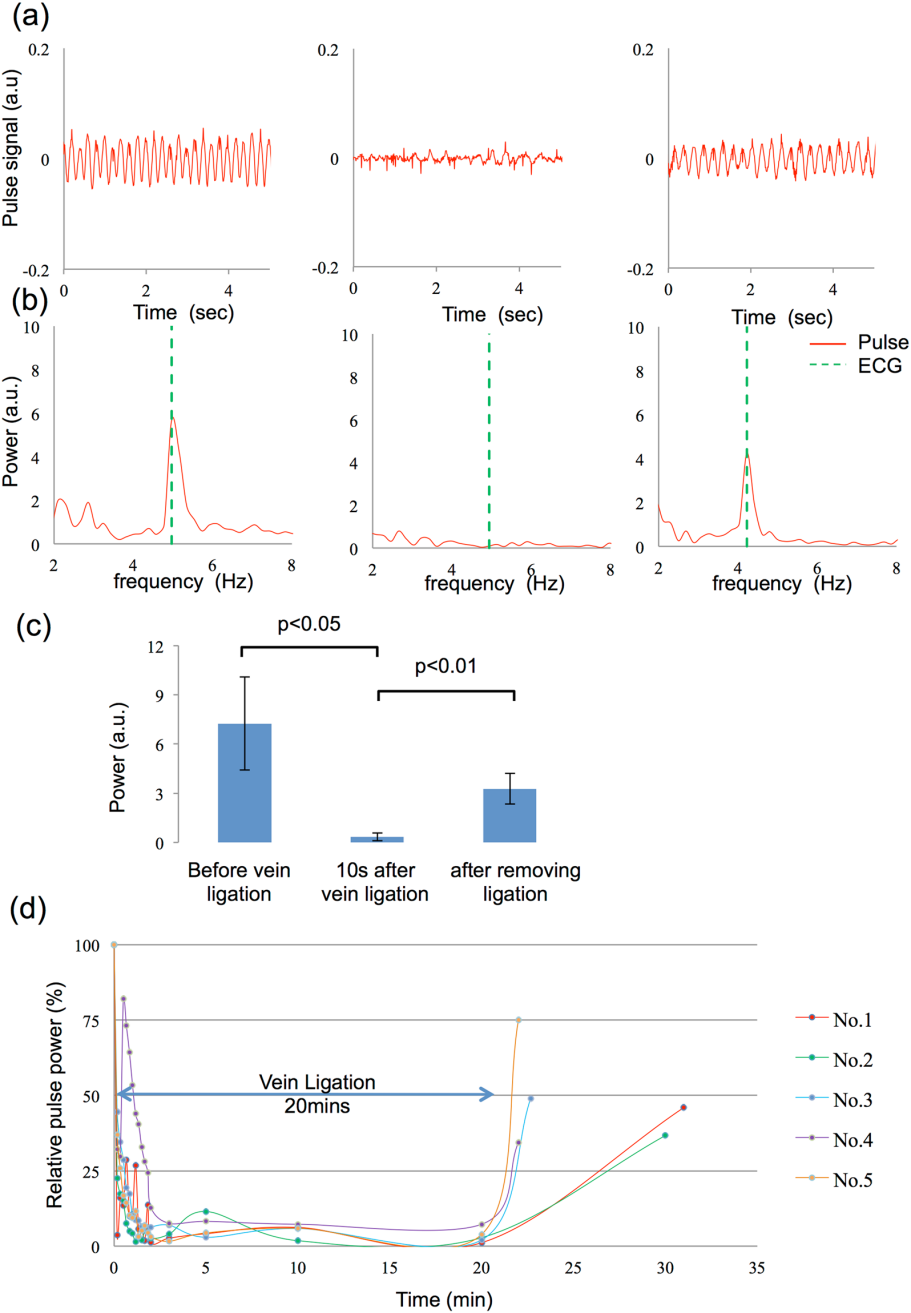
Measurement and analysis of microcirculation in the rat flap congestion model. (**a**) Data measured before ligating the superior inferior epigastric vein (left), 3 min after ligation (center), and 1 min after releasing the ligation (right). (**b**) Spectrum of pulse wave signals and electrocardiography in (**a**). (**c**) *t*-test results from 5 rats. Standard deviations in each group are $\pm$2.84 before ligation, $\pm$0.24 after ligation, and $\pm$0.93 after removing ligation. (**d**) Relative pulse power change with time. The initial value before ligation is set as a 100% reference.

Figure 3 shows the results of the congestion experiment. The left, centre, and right panels in Fig. 3a show the signals obtained before vein ligation, 60 s after ligation, and 60 s after releasing the ligation. The FFT analysis results corresponding to each pulse signal in Fig. 3a are shown in Fig. 3b. The results of the congestion model were similar to those of the ischemia model. The results of the *t*-test are summarized in Fig. 3c. A significant difference (p < 0.05) was also observed in congestion and normal blood-flow. The pulse power values of the five rats throughout the entire congestion protocol are plotted in Fig. 3d. Pulse power decreased gradually in the congestion model relative to the values in the ischemia model. Relative pulse power was maintained at <15% from 2 min after ligation, and gradually recovered to >30% after releasing the ligation.

### Pulse wave measurements in humans

Supplementary Fig. S1a shows the setup used for multipoint measurement on the human palm (See Supplementary Fig. S1). The output signals of four channels are shown in S1**b**. The results of FFT analysis are shown in Supplementary Fig. S1c. The amplitude of maximum pulse power was 600 a.u. at 0.9 Hz, while minor peaks were <100 a.u.

**Figure 4:**
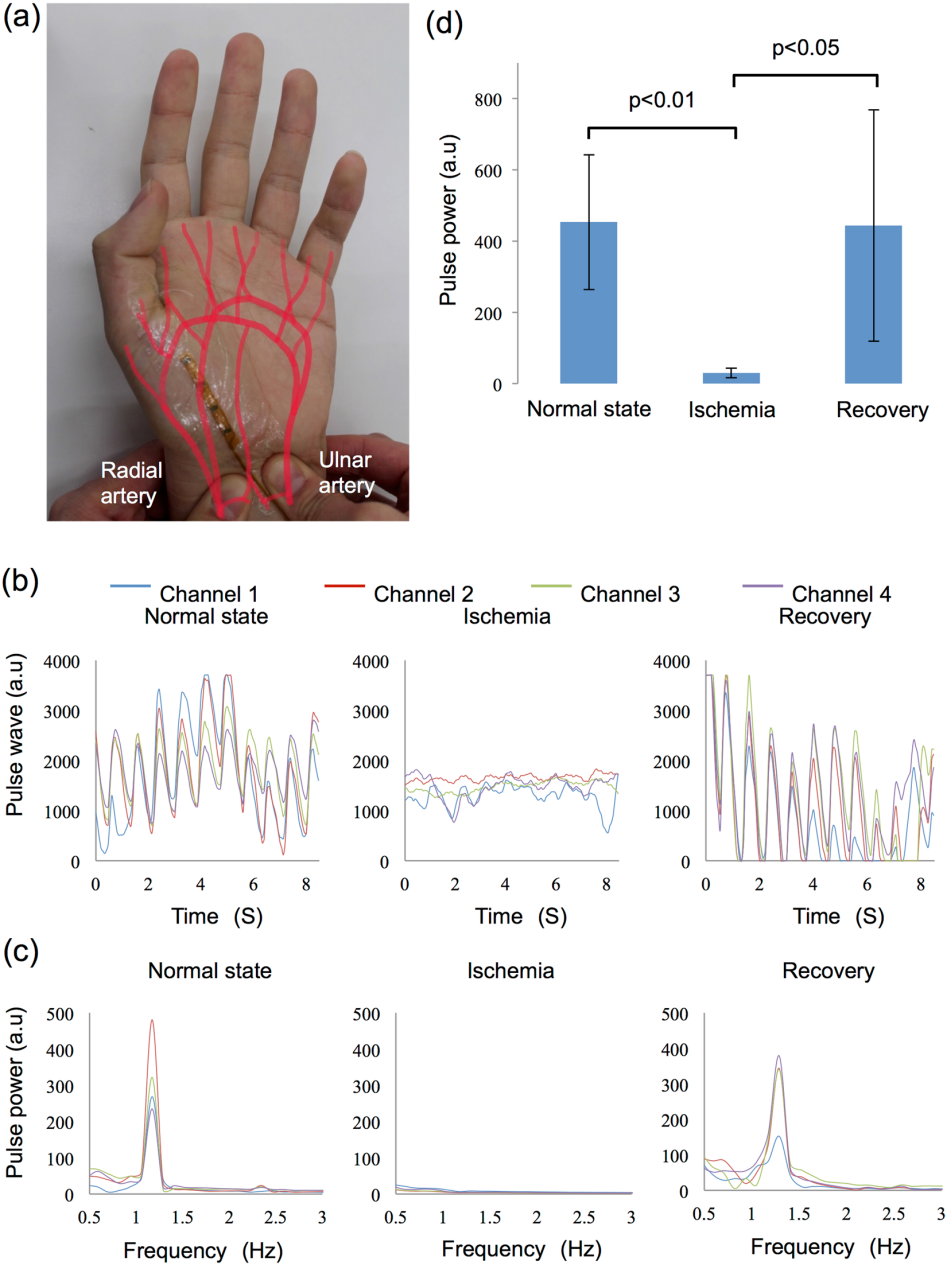
Measurement and analysis of microcirculation in a human ischemia model. (**a**) Photo of artificial ischemia on a human palm. (**b**) Data measured before pressing the radial and ulnar arteries (left), immediately after pressing (center), and after releasing the pressure (right). (**c**) Spectrum of the pulse wave signal in (**b**). (**d**) *t*-test result from five healthy subjects. Standard deviations in each group are $\pm$188.93 in normal state, $\pm$14.06 during ischemia, and $\pm$325.32 after recovery. Ligation was released after 30 s.

The sensor probe was attached to the human palm as shown in Fig. 4a during the ischemia measurements. The result obtained from one of the five volunteers is shown in Fig. 4b. Each pulse wave signal was acquired before pressing arteries (left), 2 s after pressing arteries (centre), and immediately after releasing the pressure (right). FFT analysis results corresponding to each pulse wave signal are shown in Fig. 4c. The frequencies of spectrum peaks during the normal state and recovery phase were observed at approximately 1.2 Hz, and the pulse power values were >150 a.u. as shown in Fig. 4c (left and right). However, no peak was detected during ischemia, as shown in Fig. 4c (centre). The results of the *t*-tests for pulse power among the five volunteers are shown in Fig. 4d. The amplitude of the peak was approximately 450 a.u. during the normal state and blood-flow recovery phase, and approximately 20 a.u. during ischemia. Significant differences were observed in the *t*-test results between the normal state and ischemia, and between ischemia and recovery (p < 0.01 and p < 0.05, respectively).

**Figure 5:**
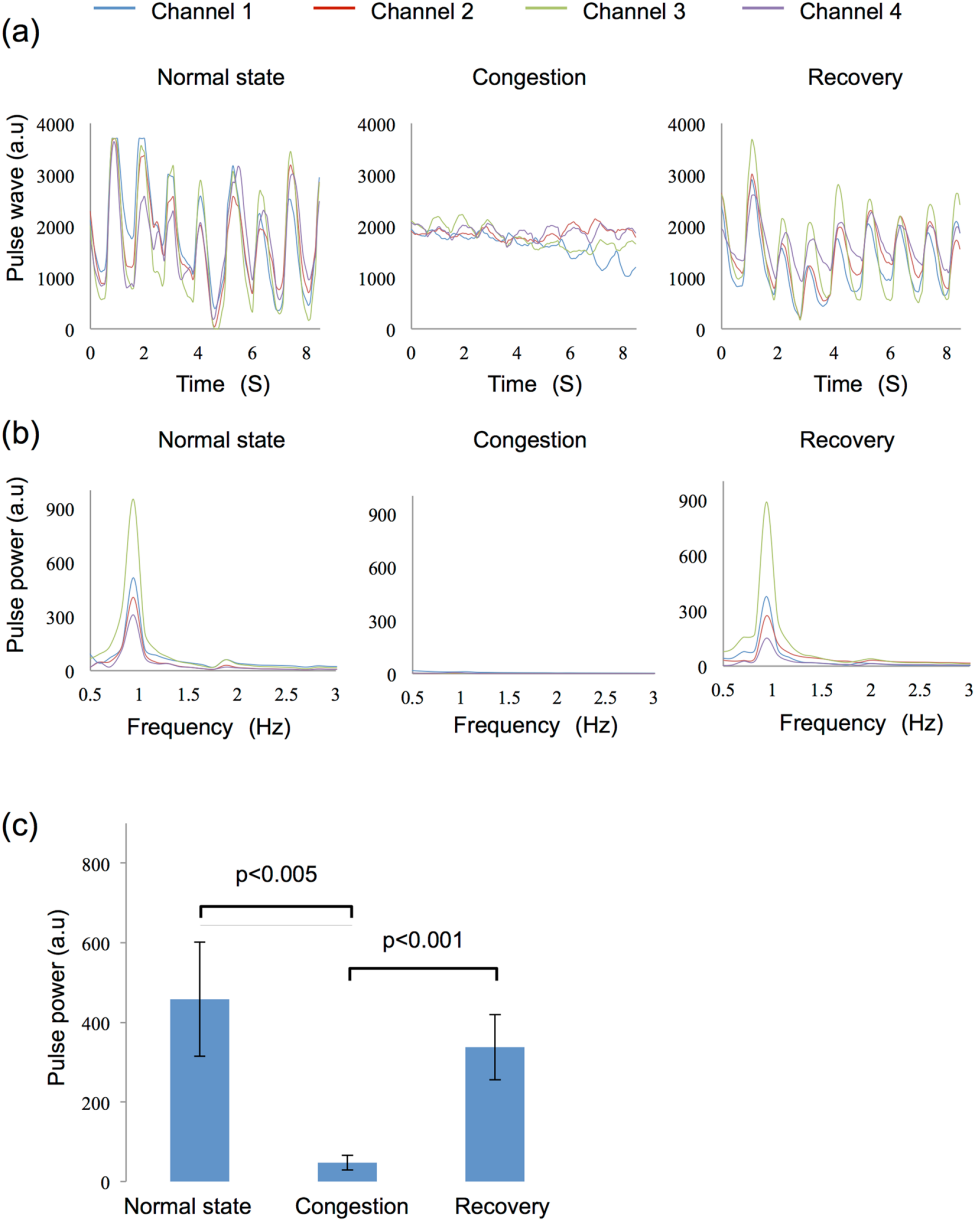
Measurement and analysis of microcirculation in the human congestion model. (**a**) Data obtained before binding the radial and ulnar veins (left), immediately after binding (center), and after releasing the bondage (right). (**b**) Spectrum of the pulse wave signal in (**a**). (**c**) *t*-test result of five healthy subjects. Standard deviations in each group are $\pm$143.68 in normal state, $\pm$18.36 during ischemia, and $\pm$82.11 after recovery. The ligation was released after 1 min.

Figure 5a shows the results of congestion measurement. Pulse wave values obtained during congestion measurement were similar to those obtained during ischemia measurement. The frequencies of spectrum peaks were approximately 0.9 Hz, as shown in Fig. 5b. The *t*-test result of pulse power among five volunteers is shown in Fig. 5c. The amplitudes of the spectrum peaks were 460 a.u., 40 a.u., and 340 a.u. during the normal state, congestion, and blood-flow recovery, respectively. The distribution of peak values among the subjects was smaller than that in ischemia measurement, and results for both groups indicated better effectiveness of pulse power in distinguishing congestion from normal blood-flow (both p < 0.005).

The results for 1 week of observation are shown in Supplementary Fig. S2 (See Supplementary Fig. 2). Blood-flow pulse wave signals were obtained for 1 min in different conditions. The pulse wave while sitting is shown in Supplementary Fig. S2a (top). The pulse wave was observed clearly with the amplitude of 1800 a.u., and the signal contained relatively little noise. The pulse wave measurement during deskwork is shown in Supplementary Fig. S2a (centre). The amplitude of the pulse wave was approximately 1000 a.u. It contained non-periodic artefacts, but was still suitable for analysis. The pulse wave while sleeping is shown in Supplementary Fig. S2a (bottom). The largest pulse wave amplitude among the three different conditions was obtained (approximately 3000 a.u.) with negligible noise. Supplementary Fig. S2b shows the results of continuous monitoring throughout 1 day. The pulse power values of all four channels were recorded successfully for 1 day.

## Discussion

There is a significant demand for blood flow measuring devices. Such devices are required after tissue transplantation and for improving the prognosis of foot gangrene in diabetes^32^. The early detection of compromised tissue circulation after tissue transplantation is critical^9, 13^. Kroll *et al*. reported that 80% of thrombi occur within the first 2 postoperative days, and that 90% of arterial thrombi occur within the first 24 h after surgery^10^. Several published reports support the notion that postoperative monitoring should be performed from 3 days to 1 week^9, 10^. Conventional check-ups, such as observing flap colour by eye or examining temperature by touch, require a significant level of skill to detect compromised circulation at an early stage. A pinprick test is capable of assessing arterial occlusion (if there is no bleeding), but it is difficult to assess venous congestion based on short-term bleeding observations. Furthermore, the fact that these assessments need to be repeated every 2 or 3 hours imposes burdens on both patients and medical staff, and the lag between flap failure and inspection reduces the rescue rate^8, 9, 13, 33^. The rescue rate significantly decreases when compromised circulation is detected 5 h after onset, which indicates the importance of immediate salvage^9^. Observation using conventional devices is associated with lags of hours due to manual operation. Therefore, a continuous monitoring device is urgently required.

Conventional monitoring approaches such as SpO_2_ devices, NIRS, laser Doppler, glucose detection, LSCI, and thermography have been reported in recent years^12, 19, 26, 34^. These approaches are effective for detecting ischemia or congestion. However, some devices fail to monitor both of these simultaneously due to limited sensitivity, while others need to be operated by experienced staff and only offer effective intermittent assessment while failing to allow continuous monitoring. In addition, most devices only detect a one-point signal, while mapping a tissue-size area is necessary. Even though the last two methods (LSCI and thermography) allow blood-flow mapping, the problem that patients need to expose their skin persists.

SpO_2_ monitors, NIRS, laser Doppler, LSCI, and glucose detection are effective for monitoring arterial occlusion. Although these methods are theoretically capable of monitoring venous congestion, the lag between decreasing outflow blood and changes in signal restrict the sensitivity of these devices. Generally, it has been reported that venous congestion is more likely to explain compromised circulation than arterial occlusion^7^. The effectiveness of blood-flow analysis to detect venous congestion was first reported by Hara *et al*.^20^. Henault *et al*. reported that analysis of glucose and lactic acid can reveal venous congestion 5.7 h earlier than the appearance of phenomena associated with occlusion^35^. However, blood-flow analysis is invasive, and still imposes burdens on patients and staff. The device proposed in this study not only detects arterial occlusion and venous congestion with a short lag time, but also measures continuously via a non-invasive approach that remedies the limitations of conventional approaches.

NIRS and SpO_2_ allow continuous monitoring, but the attachment of sensors limits their application to different body parts. Our sensor probe is expected to have good conformity to the tissue transplant on any body part because flexible devices can be attached to the skin much more securely than previous devices. Furthermore, existing NIRS and SpO_2_ monitoring devices are designed to measure only one point. Compromised circulation can occur partially in a flap. In many cases, partial circulatory failure in the flap can result in total flap loss. Therefore, multipoint mapping is required to detect partially compromised circulation as early as possible.

The sensors in our device are implemented on a flexible substrate at 1-cm intervals. Conventional devices are limited in their ability to perform multipoint monitoring because of the difficulty in making all sensors contact the skin. In this study, we fabricated the sensor array on a polyimide substrate and applied a PDMS sheet cover to ensure the stability of the final multipoint sensor. The flexible multipoint sensors produced were able to attach even to angular structures such as the finger and elbow joints. In this model, the device included 4 sensors, but the number of the sensors can be expanded as needed. The fact that it is durable enough for multipoint measurement is an advantage for detection of part of the circulation, or to map the entire circulation.

Significant decreases in pulse power within the frequency domain were observed, indicating that the output pulse wave signal is related to changes in blood flow. Thus, it is possible to produce an automatic blood-flow calculation algorithm using real-time FFT analysis. Real-time FFT analysis calculates pulse power within the frequency domain in each cycle. Compromised circulation is considered to be present when the pulse power is lower than a certain predetermined threshold. However, motion noise superimposed on compromised circulation signals can lead to a false normal reading. To distinguish motion noise, spectral analysis and determination of the SD of the pulse wave value can be effective. Because the motion noise of the subject usually induces sharp fluctuations, the SD value for the motion noise will exceed that of the pulse wave signal. Moreover, irregular motion noises are recognized by spectral analysis, and separated from the pulse wave data. In the future, we intend to develop and apply such an algorithm, and to assess its performance in a clinical study. In ischemia and congestion experiments, our device was able to detect compromised circulation immediately, without the lags common to existing methods. This is another advantage over previous devices. Because this method reflects real-time blood flow, there is no delay required for determination of metabolic effects, as is the case with other methods. Thus, assuming that this device becomes widely used, we expect the rate of rescue to increase in the future^9, 12, 13^.

Battery life is an important consideration in the design of wearable, continuous monitoring systems. In our new device, we used six dry batteries that together produced 1.5 volts while allowing mobility of the signal processor. We set the detection period to 1 min at 3-min intervals in order to preserve battery life. With this level of power reserve, it is possible to use the device continuously for 3 days after surgery, which fulfils the minimum requirement discussed earlier^9, 10^. This interval and scanning system allowed data to be collected every 4 min, which is acceptable in this context.

The reduced size of the device and prolonged battery life made it possible for the device to be portable. Moreover, the comfortable fit of the monitor, with the flexible system, did not interrupt the activity of the volunteer. In addition, wireless transmission of the data means that there is a great deal of freedom while wearing the device. This combined technology made it possible to obtain one week of continuous monitoring without interfering in the user’s daily life.

The device evaluated in this study could still be improved. First, the measurement area of the sensor could be extended by solving problems such as connections between channels and improving the construction of the sensor matrix. Furthermore, we aim to fabricate a multifunctional sensor probe to measure other factors related to compromised circulation. This could include the introduction of colour and temperature sensors to increase the reliability of the signal and further distinguish ischemia and congestion.

In this study, we developed a flexible multipoint pulse wave-monitoring device. We succeeded in the detection of compromised circulation in both ischemia and congestion models in animals and humans. Furthermore, we accomplished 1 week of continuous wireless monitoring in healthy subjects. This device should be useful in the hospital setting, and allow patients to move freely during monitoring. This is important because insufficient postoperative activity can result in other symptoms, such as deep vein thrombosis and delirium.

## Materials and Methods

Animal experiments were carried out in accordance with the institutional regulations for animal experiments based on the governmental Guideline for Proper Conduct of Animal Experiment and Related Activities, and were approved by the institutional Committee of the University of Tokyo (KA14-6). Human subjects were also in accordance with the institutional regulations based on the governmental Guidelines for Medical and Health Research Involving Human Subjects and were approved by the different institutional Committee of the University of Tokyo (KE16-24).

### Pulse wave measurement using a reflective optical sensor

We developed a pulse wave-monitoring system using multi-channel reflective optical sensors. Supplementary Fig. S3 shows the schema of the sensor and the drive circuit (See Supplementary Fig. 3). The reflective optical sensor (NJL5303R, Shinnihonmusen Corp, Japan) for each channel was composed of a green light-emitting diode (LED) (570 nm) and a phototransistor. The incident light emitted from the green LED was partly absorbed when passing through blood and tissue, and backscattered light was measured with the phototransistor and converted into electrical signals. The intensity of backscattered light varied with changes of blood volume in the capillaries. The optical signal fluctuated with the heartbeat, and the corresponding signal formed a pulse wave. Compromised circulation was detected when these fluctuations were significantly reduced. Compared to other visible and near-infrared lights, green light is absorbed more fully by blood and the signal-to-noise ratio is therefore high^36^.

### Determination of the distance between sensors

The gap between sensors should be as small as possible to increase the density of sensing points on the target tissue. However, if the gap between sensors is too small, the backscattered light from adjacent sensors will be detected by neighbouring sensors. We carried out a pilot experiment in rats to determine the optimal distance between sensors without interference. The amplitude of the pulse wave was measured by attaching two sensors to the rat skin with various gap sizes. Supplementary Fig. S4 shows the normalized amplitude of the pulse wave against the distance between the LED and phototransistor (See Supplementary Fig. 4). The reference amplitude was obtained at 1.45 mm. The interference between sensors was negligible when the distance between the sensors exceeded 3 mm.

### Flexible multipoint probe

**Figure 6:**
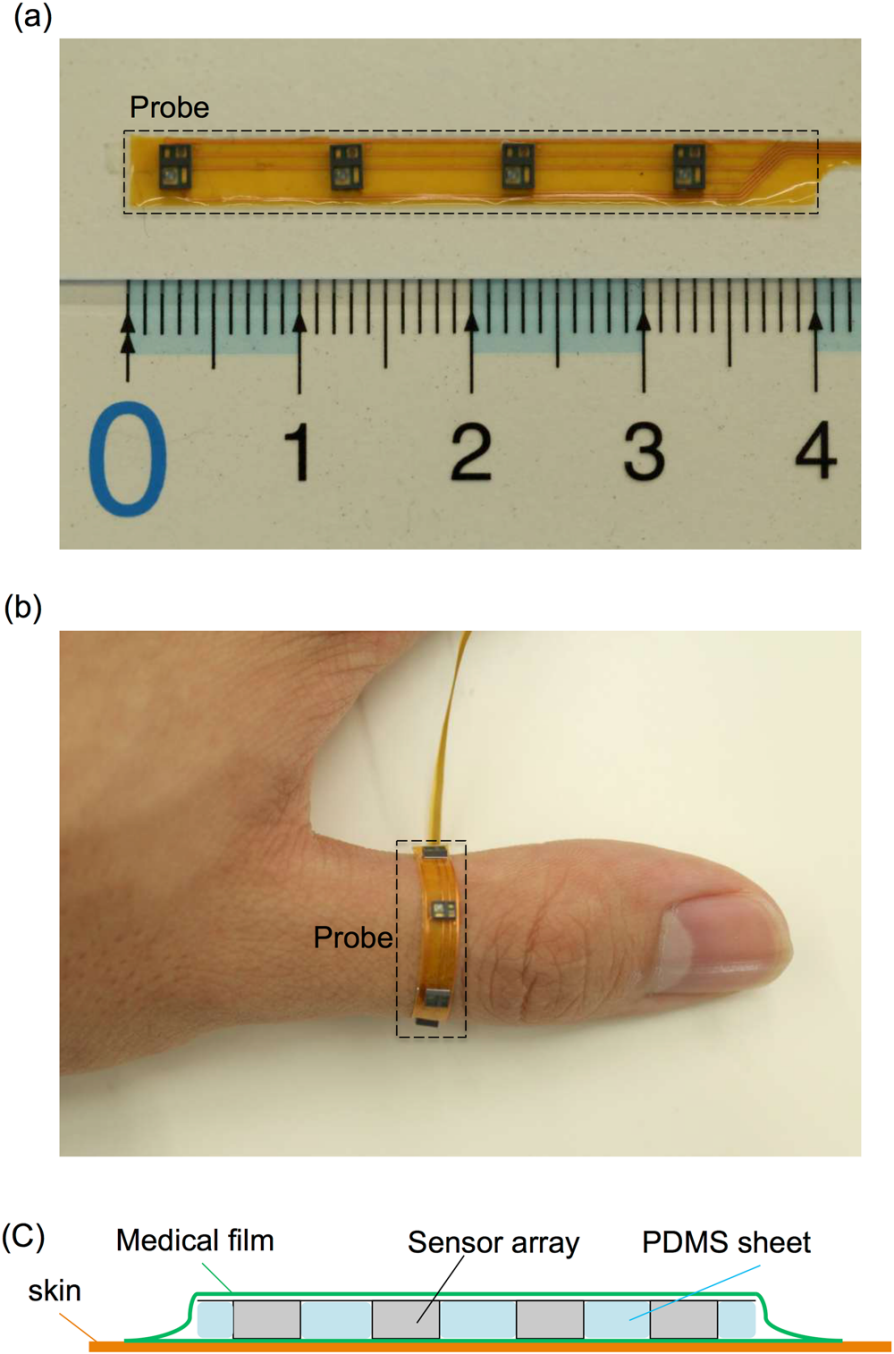
The structure of the pulse wave sensor probe. (**a**) Size of the sensor probe. (**b**) Flexibility of the sensor probe. (**c**) Structure of the sensor probe with a polydimethylsiloxane sheet.

As shown in Fig. 6a and b, we implemented four optical sensors with a 1-cm gap on a 33-µm-thick flexile polyimide substrate. The contact surface of the probe was uneven, and the angle between sensors and the skin was able to change with body movements. We fabricated a flexible and biocompatible sheet made of polydimethylsiloxane (PDMS) and combined it with the probe to produce a flat surface, as shown in Fig. 6c. As a result, the angle of optical sensors with respect to the surface of the skin was stabilized, and the robustness of measurements against body movement was improved. 7**a** shows the entire pulse wave-monitoring device system.

### Signal processing

**Figure 7:**
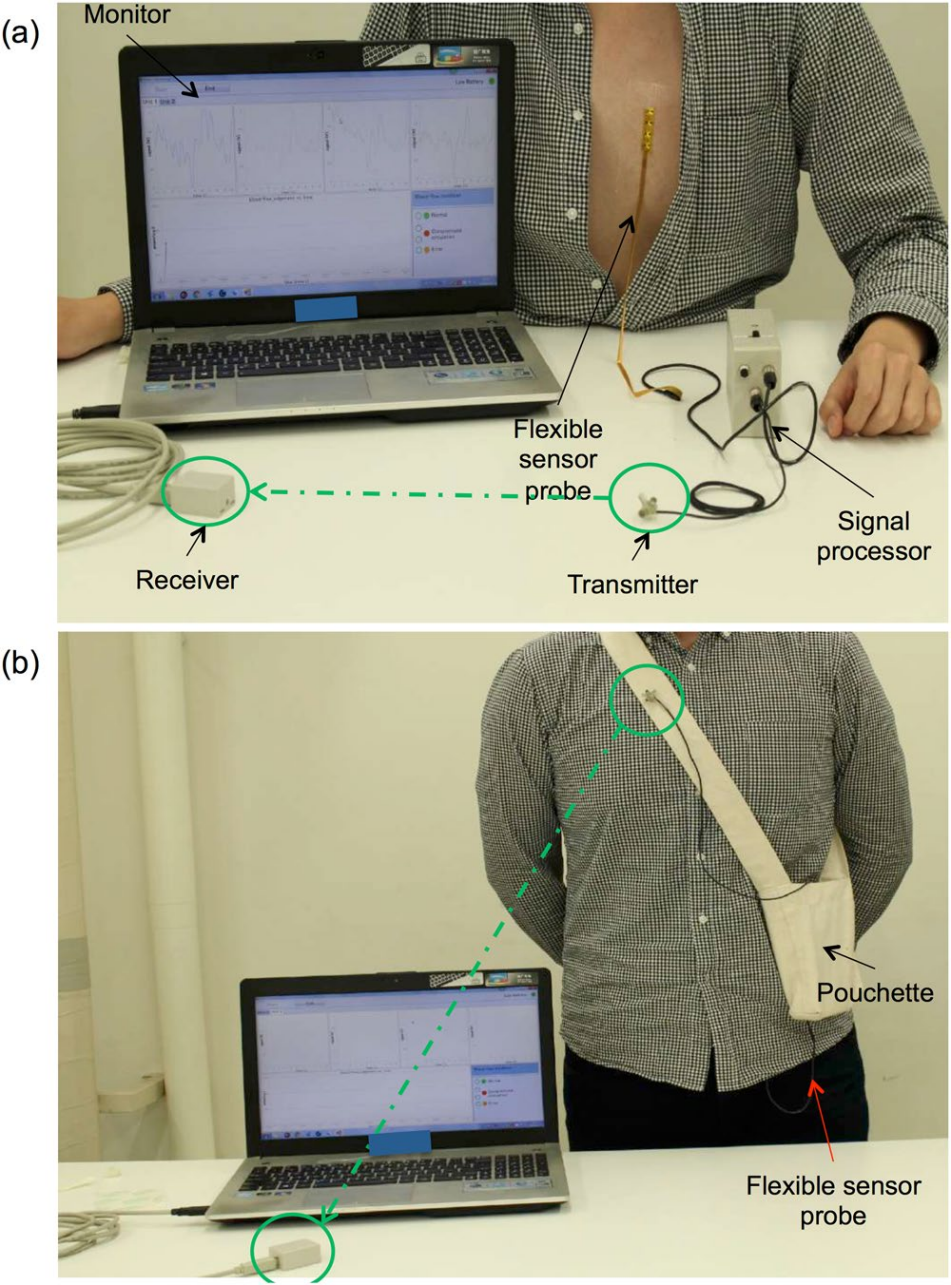
The entire monitoring system. (**a**) Attachment and setup during measurement. (**b**) Operation as a portable device: the data processor is kept in a small bag.

The output signal of the probe attached to the body included various types of noise, such as power source noise and artefacts caused by respiration and motion. Furthermore, the ratio of the pulse wave component to the DC component was only 0.3%. Therefore, we prepared a pass band filter and amplifier to extract the pulse wave component^37, 38^. The pass bands for rats and humans were 2–16 Hz and 0.1–10 Hz, respectively, due to differences in heart rate. The dynamic range of the system was 72 dB. We developed a portable signal processor that can be kept in a small bag, as shown in Fig. 7b. The signal processor was equipped with a wireless infrared device to transmit data to a PC to allow subjects some degree of movement.

### Protocol for the animal model

We demonstrated multipoint measurement of pulse waves. The probe was attached on the back of a Wistar rat (441 g) and normal pulse waves were measured for 5 s.

**Figure 8:**
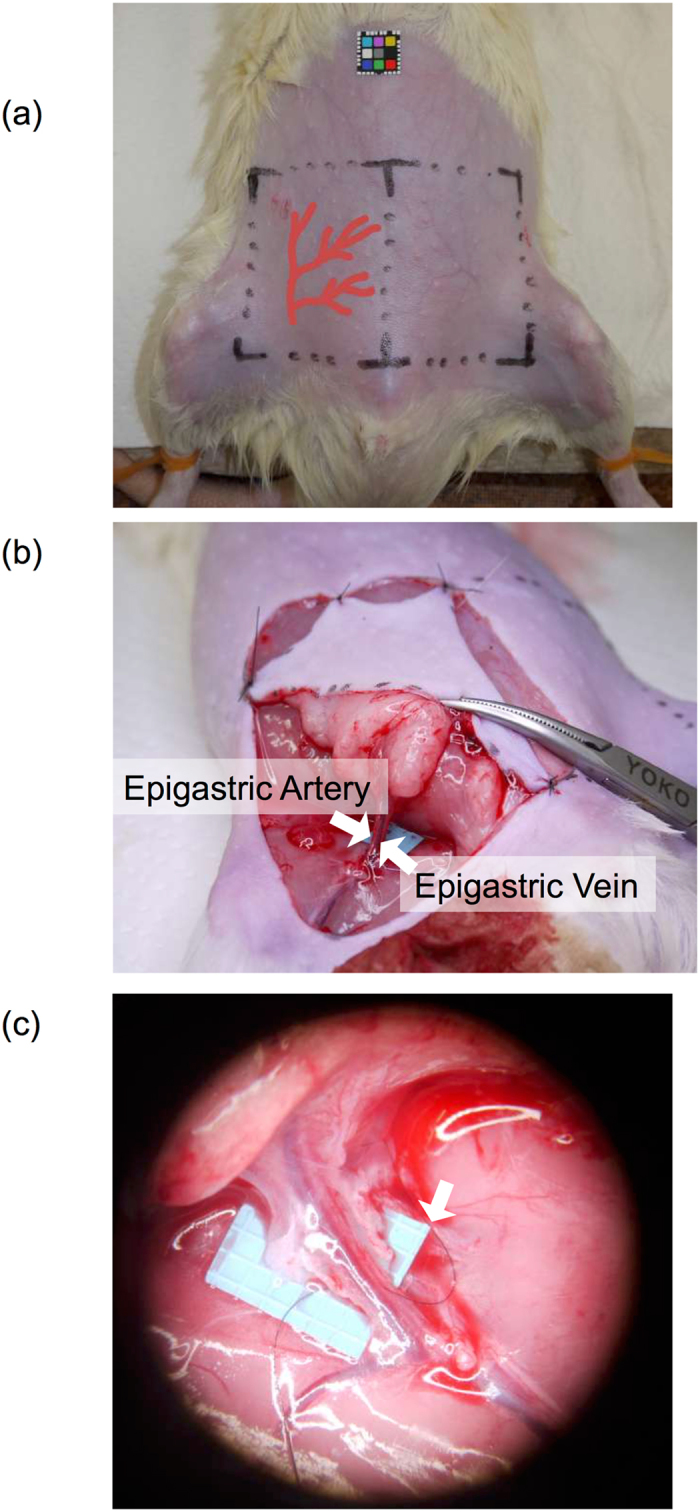
Method for creating a compromised circulation model in rats. (**a**) Area of the groin flap. (**b**) A flap based on the inferior epigastric artery and vein was raised, connected only with the pedicle. (**c**) The artery or vein was ligated with 9-0 nylon to produce the ischemic or congestive model.

To detect two major types of compromised circulation (ischemia and congestion), we carried out two experiments to evaluate the efficacy of the system. Five Wistar rats (mean ± standard deviation [SD] body weight: 408.2 ± 20.6 g) were used in the ischemia model experiment. The black line in Fig. 8a marks the left and right groin flaps based on the inferior epigastric artery and vein. The left groin flap was raised as shown in Fig. 8b, and all of the subcutaneous tissue was dissected. The flap was connected to the body only by the artery and vein, as shown by the arrows in Fig. 8b. We induced artificial ischemia by ligating the target artery using a thread (monofilament polyamide, diameter of 0.03 mm) as shown in Fig. 8c, and performed reperfusion by releasing the ligation. This procedure was performed under stereomicroscope by a plastic surgeon. The probe was covered with a medical transparent film (Tegaderm^®^ 3 M, USA) and attached to the rat. The pulse wave signal was obtained for 1 min before ligating the artery, and for 2 min after ligation. The measurement of microcirculation of the flap continued after releasing the ligation until we observed obvious recovery of the pulse wave (in <20 min). An electrocardiogram (ECG) was recorded throughout the experiment.

Five Wistar rats (mean ± SD body weight: 434.8 ± 22.1 g) were used in the congestion model experiment. The groin flap was raised, and we induced artificial congestion by ligating the target vein, similar to the process for the ischemia model. Finally, the ligation was released. The pulse wave signal was obtained for 1 min before ligating the vein and for 20 min after ligation. Vein ligation lasted longer than artery ligation because the reduction of the pulse wave amplitude was slower compared with that in the ischemia model. After the ligation was removed, measurement was continued until we observed obvious recovery of the pulse wave (in <20 min).

### Analysis for the animal model

The acquired data, including pulse wave and reference ECGs, were filtered using a finite impulse response (FIR) 3rd-order high-pass digital filter (cutoff frequency: 1.5 Hz) to remove low-frequency fluctuations caused by respiration (~1 Hz). The amplitude of the pulse wave was calculated by applying a fast Fourier transform (FFT) method. We evaluated the frequency band 4–8 Hz because the heart rate of anesthetized rats is approximately 6 Hz. The local maximum value of the spectrum was used to verify the pulse wave in the corresponding tissue. We defined this value as “pulse power”. A total of 512 points of pulse wave signal were used in FFT analysis in order to calculate pulse power, corresponding to a pulse wave signal of 5 s. The ECG reference was analysed using the same method.

The changes in pulse power caused by ischemia and congestion were assessed using a paired *t*-test on the results from five rats. The following were assessed using p-values: difference between before and after artery/vein-ligation, and difference between after artery/vein-ligation and after releasing ligation. The change in pulse power as a function of time was demonstrated by calculating the relative amplitude of the pulse wave. In the rat model, pulse power before ligation was treated as a reference, and the subsequent outputs were normalized.

### Protocol for measurements in humans

Informed consent was obtained from all volunteers. Five healthy volunteers were each asked to sit on a chair, and the probe was attached to their palm. The pulse wave obtained was regarded as the “normal state signal”.

The ischemia test began with the volunteers in the normal state. A modified Allen test, which is a common physical examination of arterial blood flow of the hand, was used to induce artificial ischemia. We instructed volunteers to clench their fingers, and then press their radial and ulnar artery to the wrist under the supervision of a physician. Reperfusion was performed by releasing the pressure. The pulse wave was measured for 1 min both before and after pressing the artery to the wrist. We measured the pulse wave for 1 min after releasing the pressure to observe the recovery of circulation.

The congestion test also began with the volunteers in the normal state. To create artificial congestion, we used a tourniquet to tie the forearm and produce pressure. Similar to a blood draw, the cutaneous vein was occluded without occluding the artery, causing congestion of the palm. Reperfusion occurred by untying the tourniquet. The measurement time in each state was the same as that in the ischemia experiment.

### Analysis of human measurements

A digital filter was not applied to the human measurements because the obtained pulse wave values had a higher signal-to-noise ratio than those obtained in animal experiments. The amplitude of the pulse wave was calculated using an FFT analysis, similar to that used in the animal studies. Some parameters were adjusted: the spectrum of 0.5–2 Hz was used since the heart rate of volunteers was distributed over 30–120 beats per minute, and a pulse wave signal of 256 points was used to calculate pulse power, corresponding to a pulse wave signal of 16 s.

As in the rat model, the change in pulse power caused by compromised circulation in the five human volunteers was assessed using a paired *t*-test. Differences between normal state and ischemia/congestion, and between ischemia/congestion and recovery were determined using p-values.

### Protocol and analysis of continuous monitoring of pulse wave on the human chest

The sensor unit was attached to the chest of one healthy volunteer for 1 week and measurements were made during all of the subject’s daily activities. The medical transparent film mentioned previously was replaced each day. Our system recorded the pulse wave for 1 min at 3-min intervals to preserve battery power. Therefore, the pulse power was calculated once every 4 min.

### Analysis of repeatability, reproductibility, and stability of the system

The output of the measurement system is pulse wave, and the amplitude of pulse wave result is represented by pulse power, which is the peak power of spectrum of pulse wave signal. Repeatability is defined as the ability to obtain same pulse power when measuring the same skin tissue by the same tester and the same device. Reproducibility is defined as the ability to obtain same pulse power when measuring the same skin tissue by the different testers and different devices. And stability is defined as the signal percentage drop in the zone of center line ± σ.

This experiment involved one healthy volunteer, and we instructed him to sit silently. The sensor probe is attached to palm of the volunteer, and we marked the contour of attaching position. In repeatability test, only one tester attached the sensor on the volunteer, and repeated for 10 times. Each measurement lasted for 1 minute, and repeatability was 77.6% (See Supplementary Fig. S5a). In reproducibility test, five testers attached the sensor on the volunteer using 3 sets of monitoring system. Totally 15 groups of data were obtained. Each measurement lasted for 1 minute, and reproducibility was 79.8% (See Supplementary Fig. S5b). In stability test, one tester attached the sensor on the volunteer, and measured data continuously for 4 hours (See Supplementary Fig. S5c). We took the log of volunteer and exclude the data when there is obvious active movement. Stability was 92.2%.

## Electronic supplementary material

Dataset1
