# Supplementary material for: Multipoint Tissue Circulation Monitoring with a Flexible Optical Probe
Source: Sci Rep. 2017 Aug 29;7:9643. doi: 10.1038/s41598-017-10115-5 (PMC5575279; doi:10.1038/s41598-017-10115-5)

Multipoint Tissue Circulation Monitoring with a Flexible Optical Probe

Yoko Tomioka, Shintaro Enomoto, Jian Gu, Akiko Kaneko, Itsuro Saito, Yusuke Inoue, Taeseong Woo, Isao Koshima, Kotaro Yoshimura, Takao Someya, Masaki Sekino

S 1. Measurement and analysis of multipoint microcirculation detection on the human palm

(a) Setup of the sensor probe. (b) The pulse wave signal obtained from all four channels (top left: channel 1, top right: channel 2, bottom left: channel 3, bottom right: channel 4), immediately after activating the device. (c) Fast Fourier transform analysis of the pulse wave signal corresponding to one channel in (b).

(a)

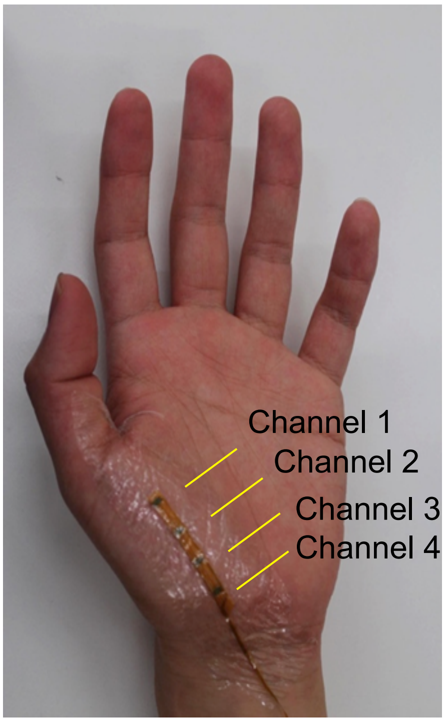

(b)

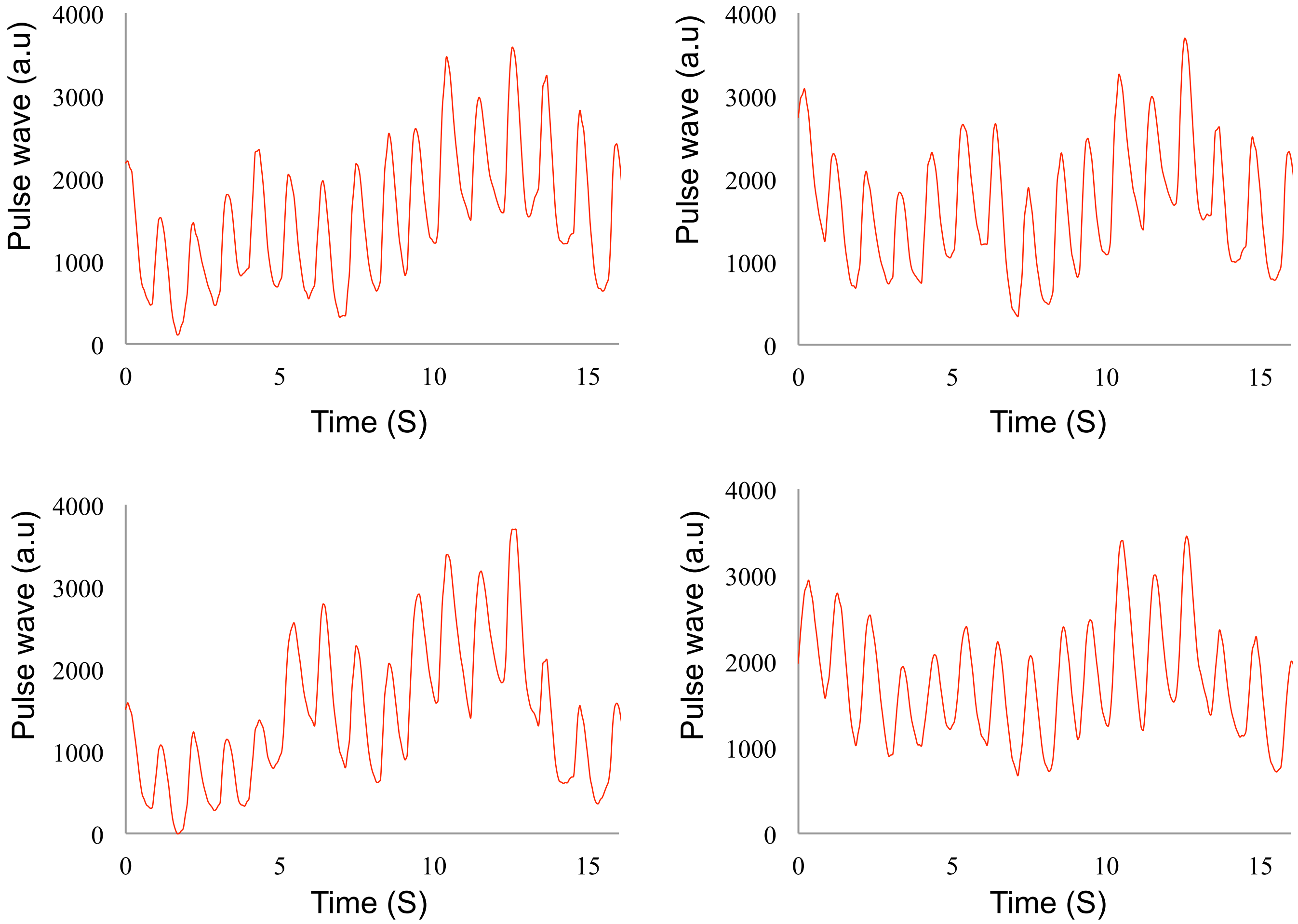
Pulse wave (a.u)
4000
3000
2000
1000
0
0
5
10
15
Time (S)
Pulse wave (a.u)
4000
3000
2000
1000
0
0
5
10
15
Time (S)
Pulse wave (a.u)
4000
3000
2000
1000
0
0
5
10
15
Time (S)
Pulse wave (a.u)
4000
3000
2000
1000
0
0
5
10
15
Time (S)

(c)

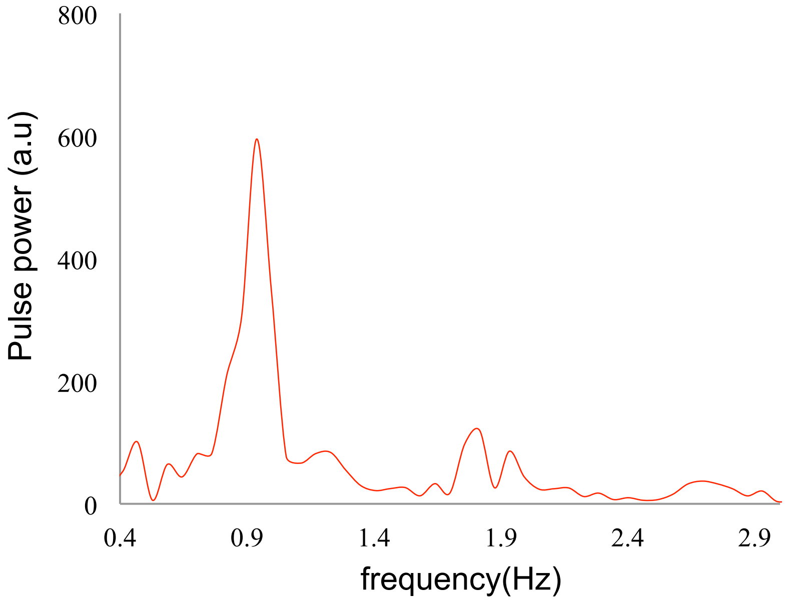
Pulse power (a.u)
800
600
400
200
0
0.4
0.9
1.4
1.9
2.4
2.9
frequency(Hz)

S 2. Continuous monitoring in a human subject

(a) Pulse wave measured when sitting quietly (top), typing (centre), and sleeping (bottom). (b) Four-channel pulse power result plotted over 1 day.

(a)

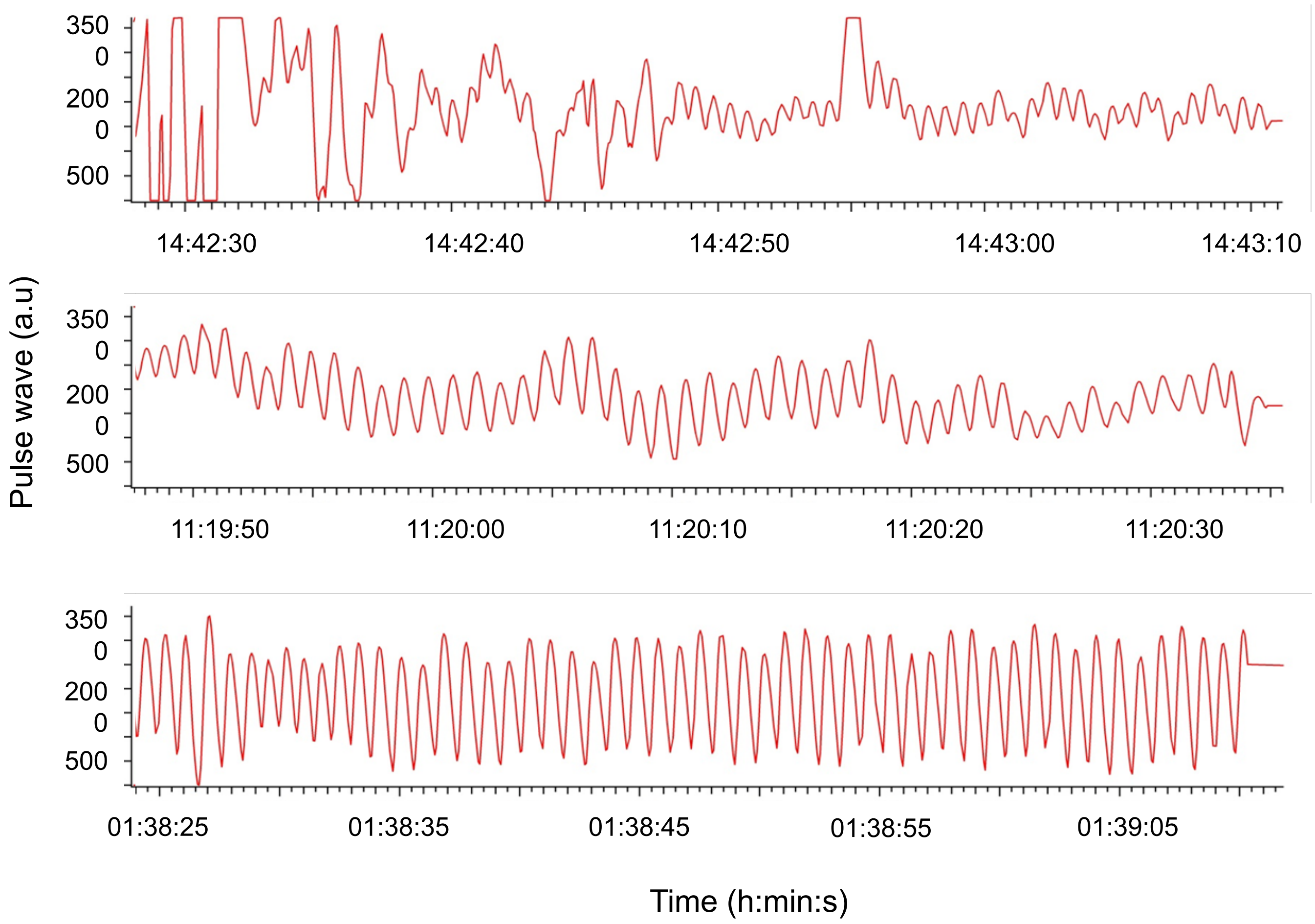

(b)

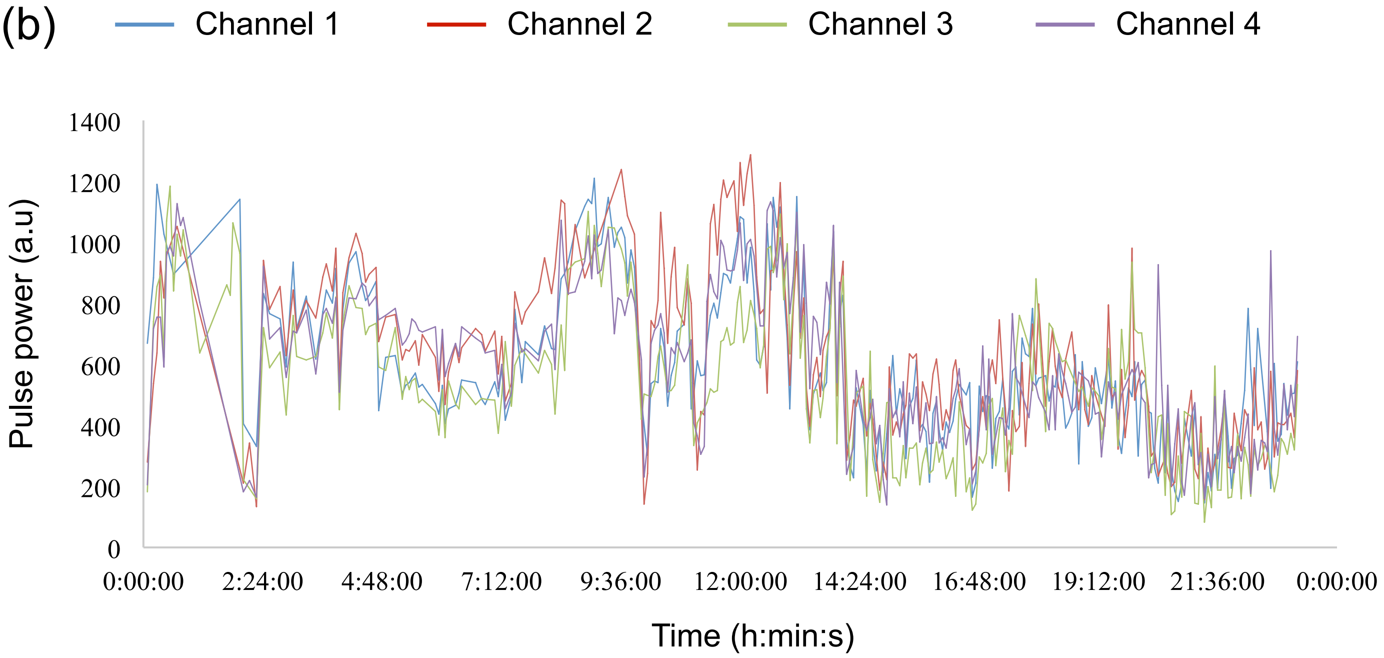

S 3. Overview of the monitoring system

The left pane shows one of four optical sensors on the sensor probe; the central pane shows the signal processor; the processed data are transmitted to a personal computer (PC).

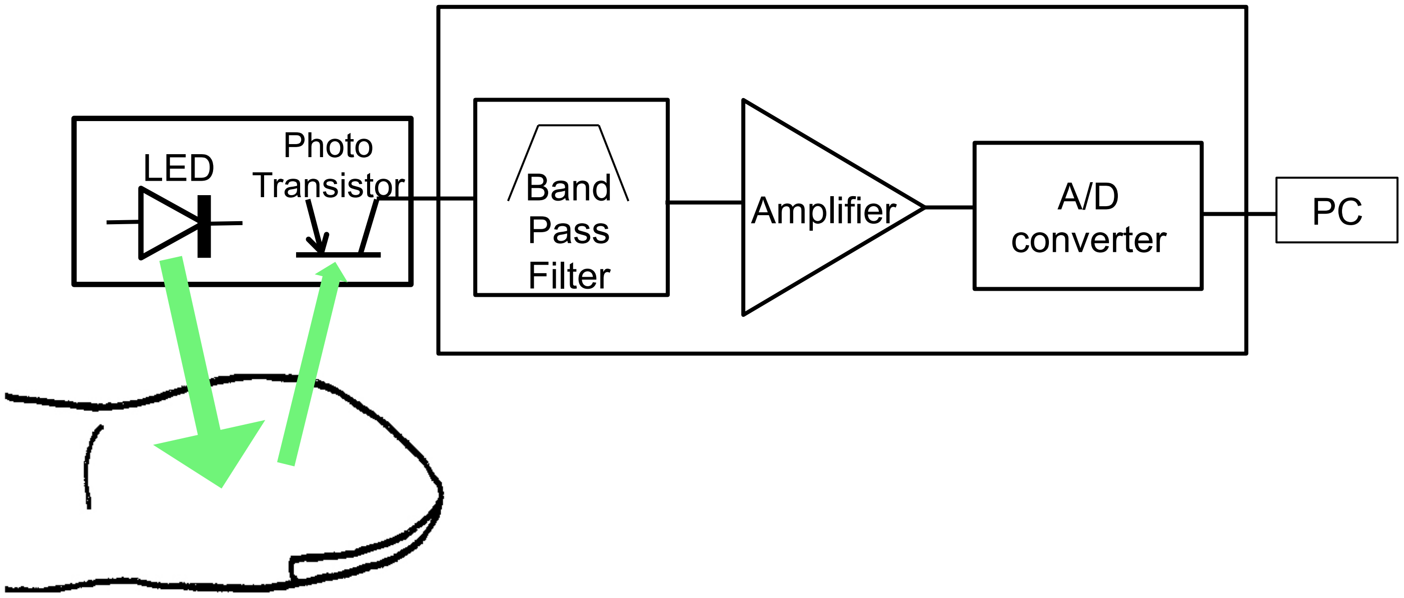

S 4. Relationship between the interval of the light-emitting diode (LED) and phototransistor and the intensity of interference

The x-axis shows the distance between the LED of the displaced sensor and the phototransistor of a fixed sensor. The error bar shows the standard deviation between 4 time measurements. Error of first two data are 7.7% and 9.3%, others are less than 1.5%.

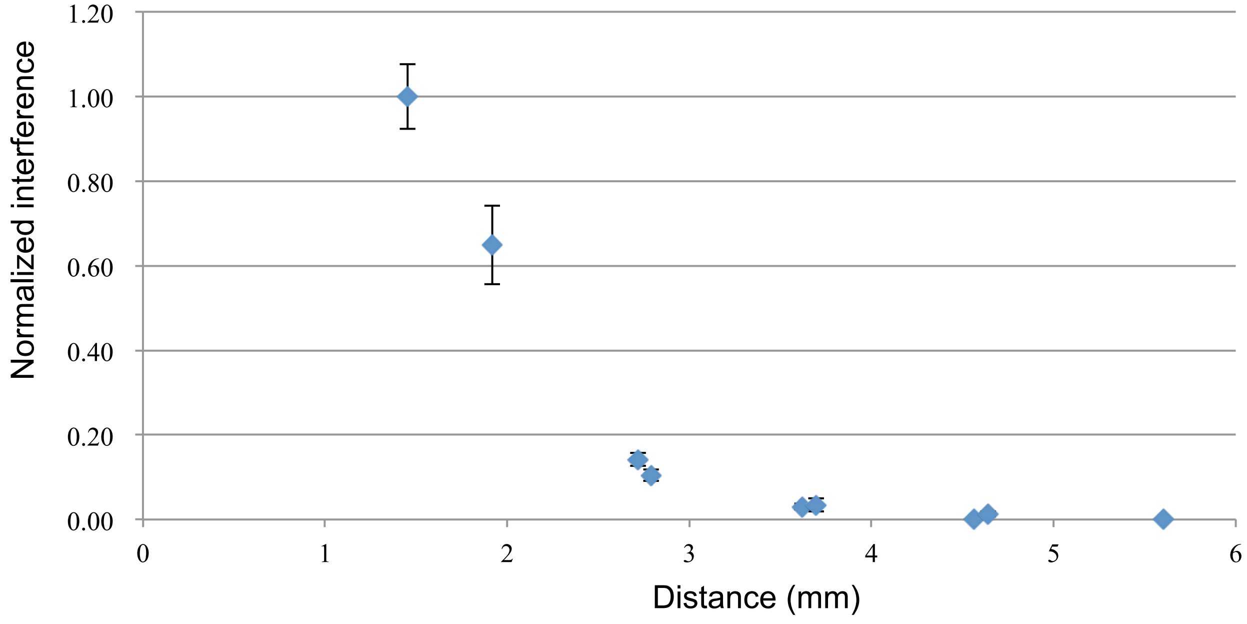

S 5. Analysis of repeatability, reproductibility, and stability of the system

(a) Repeatability was measured 77.6% by 10 repeating measurement of same object by same tester with same device. (b) Reproductibility was 79.8%, by measuring same object by 5 testers with different device. (c) Stability, which was 92.2%, was measured by 4 hours continuous measurement.

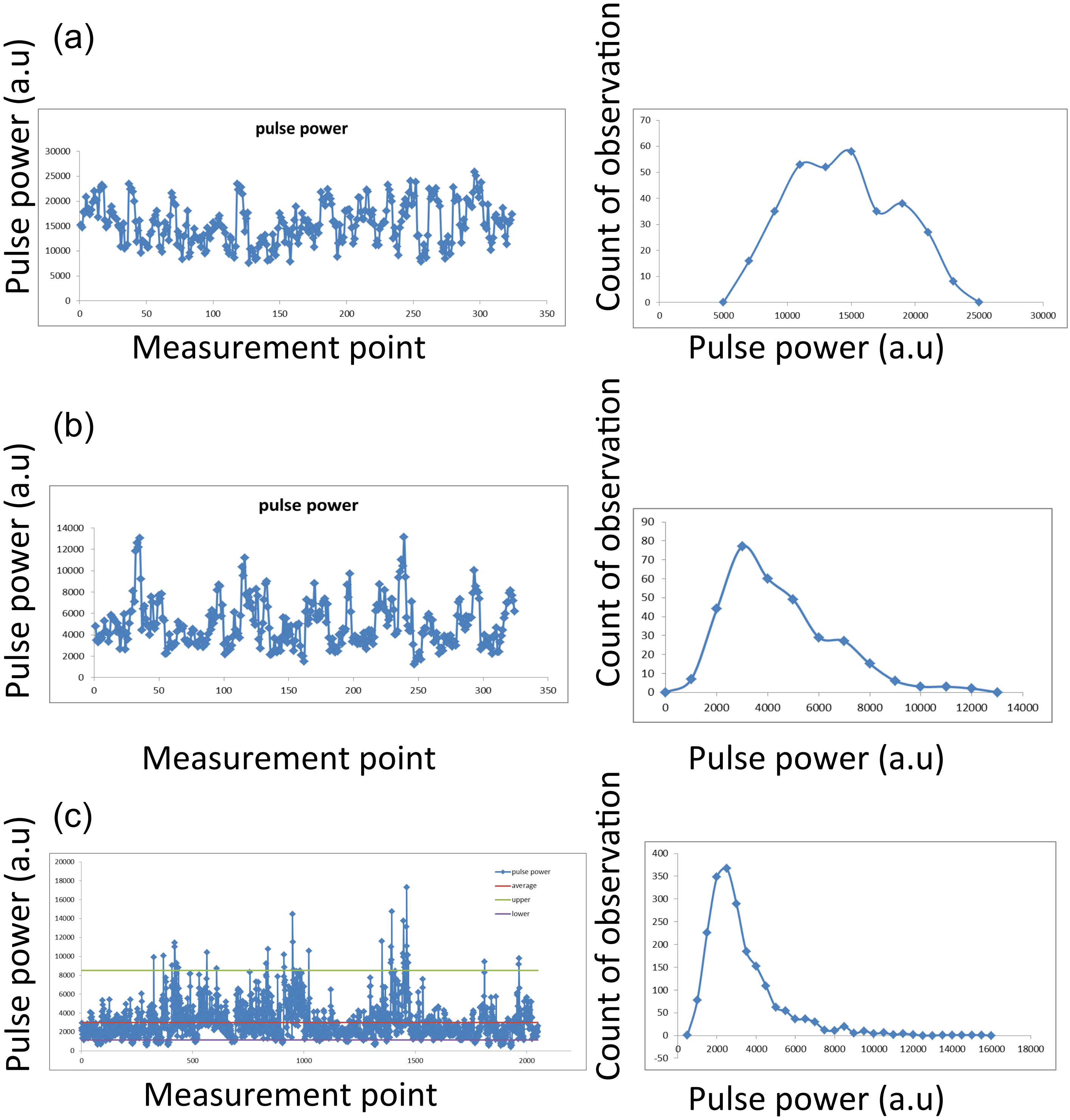

Supplement: Supplementary file 1 — Dataset1 [file 41598_2017_10115_MOESM1_ESM.pdf]
